# Surgery and technical skill decay

**DOI:** 10.1097/JS9.0000000000002313

**Published:** 2025-04-01

**Authors:** Marta de Andres Crespo, Panagis Michael Lykoudis, Fiona Myint, Pasquale Berlingieri

**Affiliations:** a Centre for Screen-Based Medical Simulation, Royal Free Hospital, London, UK; b Division of Surgery & Interventional Science, Royal Free Campus, UCL, London, UK; c Department of Vascular Surgery, Royal Free Hospital, London, UK

**Keywords:** skill decay, skill fade, technical skill, surgical education, simulation

## Abstract

**Background:**

An increasing number of surgical trainees are taking time out of clinical training for research, parental leave or other interests. A comprehensive review was carried out to evaluate the current evidence on whether and how such time results in surgical skill decay.

**Methods:**

A PubMed, Embase, Web of Science, and Cochrane Library search was performed using the phrase: (“skills decay” OR “skills fade”) AND “surgery.” All relevant literature was analyzed and summarized.

**Results:**

A total of 41 relevant articles were identified. The skills that are most adversely affected by time out of training are technical operative skills and, within those, speed and accuracy in operations. Factors that affect skill decay include the complexity of the task itself, the degree of overlearning (i.e., the skill of the surgeon prior to time out of training) and the retention interval (i.e., the length of time for which the trainee is out of training and whether or not spaced practice is carried out). The articles suggest that simulation may be of assistance in mitigating skill decay; however, this has yet to be fully investigated.

**Conclusions:**

As an increasing number of surgical trainees are taking time away from clinical training for academic research, higher degrees, parental leave, or other interests, further research is required to investigate how to mitigate the resulting surgical skill decay, potentially through the use of simulation.

**HIGHLIGHTS**

- Increasing numbers of surgical trainees are taking time out of program for research, maternity and paternity leave.
- There is insufficient understanding of technical surgical skill decay, its impact on patient safety and how to mitigate it.
- Study shows the aspects of surgical skill most affected by time away from clinical practice and the factors that influence these.
- Appropriate preventative approaches against these factors through simulation, or otherwise, may be considered in a return-to-work format.

## Introduction

Surgeons are required to undergo rigorous training and hone their skills through years of practice and experience. However, a critical concern looms over the profession. Given the increasing competitive pressures, surgical trainees are taking time out of clinical practice for academic research and higher degrees. Moreover, as society strives for equality between the sexes, more surgeons are taking parental leave, be it maternity or paternity leave. That time away from training leads to the deterioration of skills over time. This phenomenon, known as “skill decay,” has garnered increasing attention in recent years as a hiatus in training affects surgeons in a unique way when compared with other medical specialties.

Multiple, single-center studies, have found that both faculty and residents perceive an objective and subjective degree of skill decay with time out of training.^[^1-6^]^ The consequences extend beyond individual performance. Patient safety and outcomes are directly linked to the proficiency of surgeons. Studies have correlated skill decay with increased error rates and longer operative times, potentially heightening the risk of complications during procedures[7]. Such implications underscore the urgency of addressing skill decay within the surgical community.

The emergence of new technologies and techniques in surgery adds another layer of complexity to technical surgical skills. Surgeons must not only maintain their current skill-set and prevent decay, but must also adapt to innovative procedures and equipment, requiring continuous learning to stay updated[8].

Despite these challenges, efforts to mitigate skill decay have gained momentum. Interventions focussing on structured continuing education, simulation-based training and deliberate practice have shown promise in maintaining and enhancing surgical proficiency over time[9]. For instance, virtual reality (VR) simulations offer a safe environment for surgeons and surgical trainees to refine their skills without putting patients at risk,^[^10-13^]^ while regular workshops and refresher courses enable them to remain abreast of evolving techniques.

In light of the changing healthcare landscape, understanding and addressing surgical skill decay is imperative. In this article, we delve deeper into the impact that time out of training has on surgical skills. In particular, we look at which technical skills are most likely to be affected and potential methods for mitigation. We also investigate which specific factors affect skill decay and the potential to target them in the future.

## Search strategy

A thorough literature search of PubMed, Embase, Web of Science and Cochrane Library databases was carried out using the following phrase: (“skills decay” OR “skills fade”) AND “surgery” on 18 August 2024. Studies were restricted by language (English) but not by publication date or status. This resulted in 162 articles.

Included studies had to be peer-reviewed, published and original research, focussing on aspects of skill decay that specifically related to surgery. We particularly sought articles that provided data on the quantitative measurement of skill decay, and factors that influenced these rates. Review articles were excluded but their references were manually searched.

**Figure 1.**
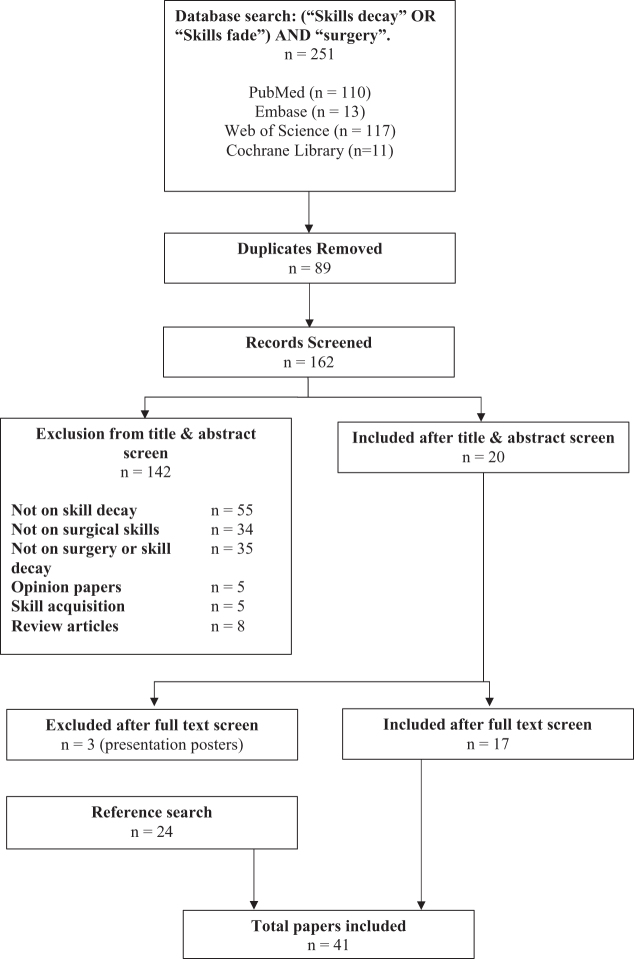
Flowchart illustrating the selection process of the studies.

The titles and abstracts were independently screened by two reviewers (MdAC, PB). Disagreements were resolved through discussion and mutual agreement. A total of 142 studies were excluded as they did not focus on the topic in question – 8 were review articles, 55 focussed on subjects other than skill decay, 34 were not specific to surgical skills, 35 were not on skill decay or surgery, 5 focussed solely on skill acquisition and 5 were opinion pieces. Full-text articles of potentially eligible studies were then assessed and a further 3 articles were excluded as they were poster presentations. Six systematic reviews were not included but their references were searched. An additional 24 studies were included as a result of this search and cross-checking references in all other included studies. The identification of the studies and its related selection process are presented as a flowchart in Fig. 1.

A final total of 41 papers were included in this review. For each study, we report the last name of the first author, the year of publication, the participants included in the study, the assessment method and procedures tested, and overall findings of the study. The data are presented in the manuscript and are summarized in Table 1.

**Table 1:** Articles included regarding surgical skills decay

Author, year	Participants	Assessment methods	Procedures tested	Findings
Akdemir, 2014 [12]	22 gynecology residents without previous laparoscopy experience.	Residents were randomly assigned (1:1) to receive training with a box trainer (1 hour per week for 4 weeks) or to a control group. At baseline and at 5 weeks, residents’ performance was assessed via the salpingectomy module of the LapSim. The box trainer group was reassessed for skills retention at 6 months.	Salpingectomy module on the LapSim.	Box trainer group performed significantly better than the control group in time and economy of movement at the final test. Error scores did not differ significantly between groups. Deterioration between final and retention tests in the box trainer group were recorded in time, instrument path length and instrument angular path but time and economy of movement scores were better at retention assessment than at baseline.
Bekele, 2019 [17]	44 medical students.	Taught instrument identification, simple interrupted sutures and one-handed knot tying then assessed at 6 weeks, 3 months, 6 months and 1 year for cohort 1 vs 6 months and 1 year for cohort 2.	Instrument identification; Simple interrupted sutures; One-handed knot tying.	At 6 months and 1 year there was a significant decrease in all three areas tested, especially practical skills of knot tying and suturing. No statistical difference between cohorts.
Bonrath, 2012 [23]	36 medical students.	Initial questionnaire.	Camera navigation; Grasping: 5 wooden beads grasped and placed in a marked field; Transfer of beads; Positioning/placement of nails on colored blocks; Pattern cutting out of gauze; Loop tie with endoloop; Extracorporeal knot tying; Intracorporeal knot tying; Clipping on rubber tubing filled with water.	Group 1: improved retention testing compared to all baseline values. No deterioration between post-test and retention test.
		Tutorial on laparoscopy and equipment handling.		
		Baseline evaluation for each task.		Group 2: no deterioration between post-test and retention test for easy (grasping and clipping), or moderately difficult tasks (camera navigation, pattern cutting) but for difficult (extra and intracorporeal knot tying) and moderately difficult tasks (transfer, positioning, loop tie), retention testing was significantly poorer. Retention test values were significantly poorer when compared to post-test values.
		Two days of training sessions with coaching and feedback.		
		Post-training testing.		
		Group 1 (retention test at 6 weeks) vs Group 2 (retention test at 11 weeks) with no practice in between.		
Castellvi, 2009 [13]	42 post-graduate year (PGY) surgical residents (year 2 to 5).	Proficiency-based training over 2 months for 5 fundamentals of laparoscopic surgery (FLS) tasks.	Peg transfer; Precision cutting; Ligating loop; Suture with extracorporeal knot tying; Suture with intracorporeal knot tying.	Task 4: retraining required for 55% of trainees after retention 1; retraining required for 40% after retention 2. Task 5: retraining was required for 86% of trainees after retention 1; retraining required for 48% of trainees after retention 2 testing.
		Follow up curriculum for tasks 4 and 5 with retention testing at 6-8 months and 11-14 months.		
D’Angelo, 2015 [1]	38 residents engaged in dedicated laboratory time from multiple general surgery training programs.	Survey prior to simulated procedures then pre and post procedure surveys to assess confidence and perceived difficulty.	Urinary catheterization; Subclavian central line insertion; Bowel anastomosis; LVH repair.	Greater reduction in technical skills and procedural steps knowledge with bowel anastomosis compared to central line and urinary catheterization. Time spent in the laboratory correlated with increased perceived reduction. Pre-procedure confidence for urinary catheterization was highest but then reduced post-simulated task. Other procedures had unchanged confidence level.
D’Angelo, 2018 [2]	66 faculty members and 79 surgical residents.	Surveys distributed to faculty and residents pre and post procedures.	Urinary catheterization; Subclavian central line insertion; Bowel anastomosis; LVH repair.	Faculty perceptions: greatest perceived reduction in surgical skills, knowledge of procedural steps and intra-operative decision making for bowel anastomosis and LVH repair. Resident survey: greatest reduction in technical surgical skills and procedural steps knowledge for bowel anastomosis and LVH repair. Gender and number of research months predicted perception of technical skills decay i.e., female with more months working in the laboratory. Comparison of faculty vs resident perceptions: significant difference with faculty perceiving less skills loss than residents.
Der, 2021 [6]	16 urologists (7 trainees and 9 experts).	Completed robotic VR exercises before and after a 6 week hiatus.	Skills assessed on robotic simulator: 1. Needle positioning; 2. Needle entry to tissue; 3. Needle driving.	Exercise completion time and instrument collisions increased. Target accuracy decreased for entire cohort, but more so with trainees than experts. Surgeons also reported a decrease in their perception of their robotic surgical skills.
De Win, 2013 [38]	145 medical students without laparoscopy experience.	Baseline assessments.	Session 1: Thumbnail – grasping, paperclip grasping, needle rotation;	1 session daily outperformed 3 sessions daily, bidaily sessions and the weekly groups. After 1 month there was still a significant advantage for regular training groups (once daily, 1 session on alternative days, 1 session weekly) over massed groups (3 sessions daily). After 6 months, the only statistically significant difference was between once daily group and three daily groups.
		Randomized into 6 groups to receive 6 training sessions of 1.5 hours each as follows: 3 sessions daily Bidaily sessions 1 session daily 1 session on alternative days 1 session weekly 1 session weekly with an optional deliberate practice in between sessions		
			Session 2 – needle positioning and penetration, throwing a surgical knot;	
			Session 3 – needle trajectory;	
			Session 4 – repeat 3;	
			Session 5 – repeat 4 but with monofilament then on chicken-skin incision to be closed;	
			Session 6 – repeat 5.	
		Retention testing at one and 6 months after final session on chicken skin.		
Gallagher, 2012 [11]	24 subjects with no prior experience in laparoscopic surgery for study 1 and 16 for study 2.	Study 1: Minimally invasive surgical trainer virtual reality (MIST-VR) used to complete 6 tasks in massed practice (all 6 tasks, 3 times, within 12 hours) or interval practice (all 6 tasks, once per day, on 3 consecutive days) or control (no training).	6 MIST-VR tasks.	Study 1: massed initially beneficial but with time, interval was better than massed and always better than control.
				Study 2: practice improved performance in terms of speed and efficiency of movements.
		Study 2: subjects randomized to practice versus no practice groups and then retested one week later.		
Guseila, 2014 [10]	11 surgical residents with no prior robotic training.	Trained to robotic proficiency with inanimate models – needle driving pad, running suture pad, ring placement on a rocking peg board.	Object manipulation; Dissection; Transection; Needle passage; Rocking peg board; Running suture pod; Tissue closure.	Residents maintained their skills for needle driving but times for suture running and rocking peg board increased by more than 20% at 8 weeks. They concluded that distributed practice was helpful but retention is selective.
		Each resident was tested on a complex tissue closure task.		
		Biweekly virtual robotic skills maintenance for 8 weeks then re-perform closure task twice with robot.		
Grober, 2004 [46]	50 junior surgical residents (PGY 1-3) were originally trained but retention only tested in 18 subjects.	Subjects were randomized to receive hands on training with bench model simulators or didactic training alone. Four months after, they were retested.	Simulators included silicone tubing or live rat vas deferens.	The retention testing, global scores and anastomotic patency rates were significantly higher for those who received hands-on bench model training compared to those who had didactic training alone. The number of interim practice opportunities with microsurgery correlated significantly with improved performance.
Grova, 2017 [5]	19 general surgery residents and graduates from UC Davis general surgery residency training program who had completed at least 1 year of research during their training.	Online survey examining factors associated with decline in skills following research years.	N/A	Majority (63%) of respondents noted a decline in their overall aptitude and technical skills during research time. Basic science researchers were more likely to report a decrease in clinical judgment. No difference between basic science and other research in terms of professionalism, communication skills. Longer time in research predicted greater decline in skills.
Hiemstra, 2009 [47]	8 medical students.	Baseline testing, five weekly training sessions and then a final test and a retention test 1 year later.	Five tasks on an inanimate box trainer Placing a pipe cleaner Placing beads Cutting a circle Knot tying Stretching a rubber band	The only score that statistically worsened at retention testing was stretching a rubber band (*P* < 0.05). All scores at retention were better than baseline test.
Howells, 2009 [32]	6 consultant orthopedic surgeons with a subspecialty interest in lower-limb surgery.	Alex Shoulder Professor benchtop simulator – performed three separate simulated arthroscopic Bankart sutures on four occasions, 1-2 weeks apart.	Arthroscopic Bankart sutures	Subjects’ performance improved with repetition. No significant retention at 6 months of the improved level of technical skill that had been acquired in the first study.
		Retention tested at 6 months.		
		Used sensors on dorsum of hand to track motion.		
Jackson, 2012 [43]	19 orthopaedic residents who have performed 20 diagnostic knee arthroscopies under supervision.	Lateral meniscus tear repair on simulator: (1) watched a video; (2) performed 12 repairs over 3 weeks; (3) randomized to either perform task once each month for next 5 months, perform task once at three months or not perform task at all; (4) final assessment at 6 months.	Knee simulator – lateral meniscus tear repair	No deterioration in skill set between the groups but those that continued to practice did continue to show small improvements in skill.
Jenison, 2012 [21]	24 attending surgeons and 27 resident surgeons who had never received formal instruction on the Da Vinci system.	Robotic surgery curriculum with didactic teaching, and completion of three modules of the proficiency test, which they repeated until they met the adjusted time required. Then repeat practice of modules at weeks 4, 8 and 12. Lastly, practice on a pig model.	3 modules:	At 4 weeks, all modules showed increase time to completion of task, after which times either decreased or stayed constant for weeks 8 and 12.
			Needle passage: pass through	
			4 entrance and exit dots;	
			Rocking ring transfer: peg transfer task;	
			Running suture: knot to be tied and then needle passed through 3 dots.	
Jones, 2017 [14]	46 residents in research.	Surveys pre- and post- simulated task in perceived reduction of clinical and surgical skills, confidence, perceived difficulty.	Urinary catheterization; Subclavian central line insertion; Bowel anastomosis; LVH repair.	Perceived skill reduction: greatest in procedure specific skills for LVH repair and bowel anastomosis, with longer time from clinical duties affecting technical surgical skills the most. Confidence: increased post-simulation for LVH repair for those participating for the first time in the study (new residents) versus a decrease in confidence post-simulation for all residents with urinary catheterization. Difficulty: pre-simulation, LVH then small bowel repair were perceived as most difficulty. After simulation, urinary catheterization showed increased difficulty as reported by new residents.
Joosten, 2022 [41]	38 medical students, medical doctors and PhD students, with no surgical experience.	Interval training for 2 tasks over 2 weeks then assigned to practice vs non-practice group and retention tested at 3 and 6 months.	Peg transfer; Interrupted suture with knot tying.	Both groups improved between baseline and post-test for both tasks. Continuous practice retained skill level after baseline whilst no practice declined at 3 months and even further at 6 months.
Kahol, 2010 [31]	10 postgraduate year 1 residents.	Baseline scores then remeasured every month for 6 months.	Virtual ring transfer with 8 cognitive variations of the task.	Skill scores for all skills showed significant deterioration from fourth month onward. Number of practice sessions required to regain baseline scores was significantly less than required to achieve initial baseline skill.
Kraemer, 2024 [19]	46 hospital corpsmen who were enlisted medical specialists in the US navy.	Participants underwent cricothyroidotomy simulation training then were randomized into one of three cohorts and asked to return only once for re-testing at 6, 12 and 24 months. Those at 24 months had one skill refresher training session.	Cricothyroidotomy.	All three cohorts experienced a significant increase in time to complete the procedure compared to initial training. There was a decline in checklist scores for 6 and 12 months but not so for 24 months. Overall skill retention (percentage of subjects who achieved performance criteria) was 31.82% at 6 months, 14.29% at 12 months, 60% at 24 months.
Linsk, 2018 [44]	24 medical students.	Randomly assigned to FLS, virtual basic laparoscopic suturing trainer (VBLaST) or control. First two groups had training over three weeks between pre- and post-tests; control had nil training.	Pattern cutting task on FLS and VBLaST.	No skill decay in any method and improvement seen with training between pre- and post-test surveys.
Maagaard, 2011 [34]	9 trainees and 10 senior consultants in obstetrics and gynecology where novices had experience of <5 procedures and experts >200.	Each participant went through 10 sessions. Session 1-3: familiarization; session 4-10: assessment.	Ectopic pregnancy module (salpingectomy).	Novices showed retention of skills after 6 months After 18 months, novices’ laparoscopic skills had returned to the pre-training level. Experts showed consistent performance over time.
		Novice group retested at 6-18 months; expert group at 6 months. None of the novices performed laparoscopic surgery in the follow up period whilst experts continued their daily work in laparoscopic surgery.		
Mashaud, 2010 [36]	91 PGY 1-5 residents were enrolled in initial FLS training curriculum; retention at 1 year was analyzed for 42 trainees; retention at 2 years was analyzed for 33 trainees.	Participants underwent proficiency-based training on all FLS tasks then enrolled every 6 months in an ongoing training curriculum that included retention tests and mandatory retraining to proficiency if not achieved.	Retention testing involved the two most complex tasks:	High retention rates for all tasks over 2 year period (92% retention for all 5 tasks).
			Suturing with extracorporeal knot; Suturing with intracorporeal knot.	
Maubon, 2022 [15]	232 UK ophthalmologists with a surgical hiatus of at least 8 weeks, prior to return to cataract surgery.	Survey distributed by the Royal College of Ophthalmologists to all non-retired UK members on 2020.	N/A	Skill decay:
				Only 11.6% had a formal plan made prior to return to surgery. 29.1% had difficulties on returning to surgery with main incision, capsulorrhexis construction, phacoemulsification and posterior capsular rupture management being the most frequently reported difficulties. Operating difficulties were related with trainee status but not gender. Perceived transient anxiety, increase in operating time and reduced confidence in operating ability – more common amongst female trainees.
				Skills reacquisition:
				Female surgeons and trainees were significantly more likely to report resource availability and to have accessed resources, e.g. simulator and online videos.
Mitchell, 2011 [42]	24 surgical interns (general surgery, urology, orthopedics, neurosurgery, noncategorical).	Randomly assigned to weekly training for 4 weeks or monthly training for four months with equal total training times.	Vascular anastomosis module with:	No statistical difference in surgical skill acquisition and retention between the weekly and monthly scheduled groups, as measured by procedural checklist scores, global rating scores of operative performance, final product analysis and overall performance or assessment of operative competence. No deterioration seen in either groups.
			Initial video of principles; Introduction to instruments; Practice on Penrose drain; practice on a graft.	
		Performance was assessed before training, immediately after, after the completion of distributed training and 4 months later.		
Molinas, 2016 [24]	73 gynecologists (34 residents and 39 specialists)	Four groups depending on number of repeats of task:	Grasp and transport 6 objects, matched by color; Intra-corporeal knot tying.	All groups retained their skills almost at the same level than after training testing with group 4 being the worst as they had no training of the task (other groups repeated it 60 times) for hand-eye coordination. For knot tying, only the first group maintained skills – they repeated each task 60 times over the course of one month.
		Group 1: repeat each task 60 times, task 1 then 2;		
		Group 2: repeat task 60 times, task 2 before 1;		
		Group 3: repeat task 2 only 60 times;		
		Group 4 no repetition.		
		Retention was tested after two years.		
Nielsen, 2023 [18]	82 medical students.	Taught emergency cricothyroidotomy and tested at 1, 3 or 6 months.	Used an evidence-based, structured assessment tool of technical skills performance to evaluate physician’s skills in the rapid four-step technique procedure with a pass/fail standard.	Significant decay after 1 month with only 6 participants passing at 1 and 3 months but nil passing after 6 months.
Nofi, 2022 [4]	76 surgical residents and 15 anaesthesiology or podiatry residents; and 34 surgical faculty with 2 anaesthesiology or podiatry faculty.	Web based cross-sectional survey.	N/A	The pandemic resulted in a reduction in case volumes and technical skills but an improvement in critical care skills. 71% of residents thought their operative training was negatively impacted by the pandemic.
Nofi, 2023 [3]	49 surgical residents and 6 anesthesiology residents; 26 surgical faculty and 7 anesthesiology faculty.	Web based cross-sectional survey.	N/A	Residents and faculty reported a reduction in resident technical skills, with a minority reporting a continued reduction at 1 year. Confidence was similarly affected. Residency year or specialty not predictive of persistent decay at 1 year.
Nofi, 2023 [26]	8 research residents who had a gap of 2-4 years from clinical training. They could moonlight on surgical services but have performed zero operations.	Enrolled in surgical rehabilitation program:	Core and advanced operations focussed on a particular surgical area (e.g. trauma, minimally invasive surgery and surgical oncology and hepatobiliary surgery).	Laparoscopic skills: performed similarly to level matched clinical residents on basic tasks but required statistically significantly more time for suturing-based skills. Research residents had significantly lower confidence levels pre-cadaver sessions but gained confidence after the session whilst clinical residents’ confidence was unchanged. 87.5% of research residents endorsed the perception of skill decay over their research years with 50% emphasizing a decay in their confidence. All residents believed simulation was an important tool in maintaining surgical skills. Benefits of simulation included: performing new cases, building confidence, receiving feedback, improving dexterity and spatial awareness and identifying personal deficiencies. All would continue to attend surgical rehabilitation workshops.
		12 cadaveric simulation sessions Fundamentals of laparoscopic-based simulation workshops of 90 minutes		
Rahimi, 2023 [16]	29 surgical residents.	Three week at-home laparoscopic box training of tasks. Then one training day 4-6 months after the course – laparoscopic cholecystectomy and appendectomy on cadavers.	Laparoscopic box tasks:	Increase in time for completion of tasks at 4 months. Worse tissue handling for post and sleeve. Increased path length for both tasks but still improved compared to baseline.
			Zigzag loop: thread through hoops in zigzag fashion; Post and sleeve: hoops on pegs.	
Rosenthal, 2010 [33]	21 medical students.	Completion of structured proficiency-based training curriculum on all five FLS tasks over 2 months with pre and post-testing. Retention testing at 6 months and 1 year.	All five FLS tasks:	Significant improvement at post-test as none had proficiency at pre-test. Retained high level of performance at retention 1 (93% of post-test score). Retained high level of performance at retention 2 (95%) of post-test score).
			Peg transfer; Precision cutting; Ligating loop; Suture with extracorporeal knot tying. Suture with intracorporeal knot tying.	
Schumm, 2022 [29]	233 general surgery residents.	Survey regarding demographics, program characteristics, impact of research and preparedness to resume clinical training and operative autonomy.	N/A	Resident satisfaction: majority (84.1%) satisfied with timing of dedicated research training. Research had a positive impact on resident development and future career, with only 11.8% reporting they would skip the research time. Resident preparedness for return to clinical work: 64% reported feeling prepared, with 16% feeling extremely prepared. Moonlighting or call experience was not associated with perceptions of preparedness. Operative autonomy: autonomy declined during research time but residents more advanced with their training were more likely to still report autonomy for basic procedures.
Sinha, 2008 [28]	33 surgical residents in general surgery from first, second and third years of training.	Trained to established criteria for 7 VR technical skills. Retention testing at 6 months with available simulators in between but no enforced practice.	Camera navigation; Instrument navigation; Camera/instrument coordination; Grasping; Lifting and grasping; Cutting; Clip applying.	Passing rates: worse for complex tasks, cutting and clip applying at 6 month retention testing. Related to seniority of resident. Path length correlated with efficiency and ability to pass session.
Spruit, 2015 [39]	41 medical students.	Group 1 received three, 75 minute training sessions on one day.	Stretch a rubber band around a set of 12 spikes; String a pipe cleaner through a set of four rings; Placement of small beads on a pegboard; Circle is cut in a rubber glove;	Outcome measures indicated better performance at the end of training, at a 2 week delayed retention session and at a 1 year retention session for the group that received training on a spaced schedule. Spacing effect was most pronounced for the more difficult laparoscopic training tasks such as intra-corporeal suturing.
		Group 2 received one, 75 minute training session per week for three consecutive weeks.		
		Retention was assessed at 2 weeks and 1 year after completion of training.		
Stefanidis, 2005 [20]	14 surgery residents with no previous VR and videotrainer simulator experience.	Subjects practiced 12 MIST-VR and 5 video-trainer tasks but retention was tested with two skills. Post-test assessment was taken after 13.2 ± 11.8 days and retention at 7 ± 4 months.	Manipulate diathermy; Bean drop.	There was an early performance decrement at post-test but the acquired skill was maintained up to the end of the follow-up period. Skill retention was better for video-trainer simulator compared with VR.
Stefanidis, 2006 [40]	18 medical students.	Trained to proficiency on the FLS videotrainer.	Laparoscopic suturing – used a 7 inch 2-0 silk suture on a tapered needle to tie a surgeon’s knot then two squares. Proficiency when score was <512 on 2 consecutive attempts.	Good skill retention during follow up across both groups. At 6 months, the ongoing training group showed better skill retention and a trend for achieving the proficiency level more often than the control group
		Then randomized to a control group who received no additional training and an ongoing training group which trained to proficiency at 1 and 3 months after testing.		
			Retention tested by performing task 3 more times.	
		Simulator testing was repeated at 2 weeks, 1 month, 3 months and 6 months after initial training.		
Stefanidis, 2008 [48]	15 novices.	Randomized to training versus control group. Training group practiced to proficiency on the FLS suturing model.	Laparoscopic suturing – used a 7 inch 2-0 silk suture on a tapered needle to tie a surgeon’s knot then two squares. Proficiency when score was <512 on 2 consecutive attempts.	Training outperformed controls. Performance of trained individuals deteriorated slightly between post-tests and retention tests on the simulator but not in the operating room; simulator training resulted in durable improvement in operative skill of trainees even in the absence of practice for up to 5 months. Porcine model had inferior performance scores than simulator with longer task times and more errors.
		The performance of both groups was assessed on the simulator and on a live porcine laparoscopic Nissen fundoplication model at training completion and 5 months later.		
Varley, 2015 [30]	30 laparoscopically naïve medical students.	One afternoon training session on skills in single-incision laparoscopic surgery and performance of tasks. Survey prior to training.	Skills in single-incision laparoscopic surgery (SILS) – needed to perform peg transfer and precision cutting as per McGill Inanimate System for Training and Evaluation of Laparoscopic Skills (MISTELS) program	Improved outcomes after retention period of 4 weeks (statistically significant) but not a significant difference at 12 weeks.
		Group A had retention testing after 4 weeks and group B after 12 weeks; no access to simulation in between.		
Van Bruwaene, 2013 [45]	39 medical students.	All students performed proficiency-based laparoscopic suturing training; then divided into four maintenance groups:	FLS suturing training Porcine Nissen model	Groups 2 and 3 significantly outperformed groups 1 and 4 on the box trainer. No difference was detected between groups on the Nissen model. Group 3 reached proficiency more quickly than the other groups.
		Control, no additional training; Massed training with one supervised training session at 2.5 months;		
		Distributed training – five monthly unsupervised sessions of 30 minutes or		
		On the LapMentor. Retention testing after 5 months: suturing on a box trainer and on a cadaver porcine Nissen model.		
Windsor, 2005 [25]	10 junior surgical registrars with little to no experience in laparoscopic surgery.	Two simulated surgical tasks repeated until no further improvement in the score for three consecutive attempts.	MIST-VR:	Each novice surgical trainee improved their score during each training session and for each task. Best score was achieved with fewer attempts during second training session. Acquisition of skills was similar across both tasks. The loss of psychomotor skills between the two training sessions was significantly different for the two tasks. There was a 23% loss for stretch diathermy task compared to an 81% loss for MD task. Reacquisition was significantly greater with manipulation diathermy than stretch diathermy.
			Stretch diathermy – stretch a cylindrical object with one hand and then apply diathermy. Manipulation diathermy – grabbing a sphere and applying diathermy to targets.	

FLS: fundamentals of laparoscopic surgery; LVH: laparoscopic ventral hernia; MISTELS: McGill inanimate system for training and evaluation of laparoscopic skills; MIST-VR: minimally invasive surgical trainer virtual reality; N/A: not applicable; PGY: post-graduate year; PhD: Philosophiae Doctor; SILS: single-incision laparoscopic surgery; UC: University of California; US: United States; VBLaST: virtual basic laparoscopic suturing trainer; VR: virtual reality; vs: versus.

Data was extracted and organized thematically into three main categories: (a) aspects of surgical skills most affected by time out of training; (b) factors affecting the rate of skill decay, and (c) strategies to mitigate technical skill decay. Where possible, we included quantitative data but given the heterogeneity of the study designs and outcome measures, meta-analyses were not performed. The study was retrospectively registered on the Research Registry (UIN: research registry 11031 from https://www.researchregistry.com/browse-the-registry#home/registrationdetails/67ad9922054efd0306f78c7b/).

## Aspects of surgical skills most affected by time out of training

### Technical skills

There are many facets to being a skilled surgeon. Perhaps the most obvious are the technical skills required to conduct an operation safely and in a timely manner. But surgeons must also be able to round on patients on the ward, provide adequate peri-operative care and manage a deteriorating patient. In addition, they must be capable to review patients in outpatient settings, make decisions on their management, and complete the necessary administrative documents needed to organize outpatient investigations and treatment. Time out of training has the potential to impact any, if not all, of these skills. However, across 12 studies, there is evidence to suggest that technical skills, namely those directly involved in procedures, are most affected by time out of training^[^1-5,14-20^]^.

Immediately after taking time out of training for research, residents report that their primary concern in terms of skill decay is a deterioration in their knowledge of surgical procedures, rather than their technical ability[14]. However, as the length of time out increases, their concern regarding declining technical skills grows, to a point where, one year into their time away, residents’ largest reported reduction in global clinical skills concerns their technical surgical skills. This is true for a variety of skills including those involved in simple procedures, such as urinary catheterization and subclavian line insertion, to more complex procedures, such as bowel anastomosis and laparoscopic ventral hernia (LVH) repair^[^1,5,14^]^. Indeed, when faculty were also involved, the majority either agreed or strongly agreed that residents returning from dedicated research fellowships demonstrated less technical skill and less confidence, and required more instruction[2].

Nevertheless, this reduction in technical skills can be recovered with dedicated clinical practice or a refresher session^[^3,4,19^]^. Coronavirus disease 2019 (COVID-19) provided a clear scenario where time out of training was enforced due to redeployment. A large cohort of surgeons suffered skill decay, which they had to recover later. Nofi *et al* investigated surgical skill decay immediately after the COVID-19 pandemic redeployment and 1 year after resumption of normal clinical work^[^3,4^]^. In the initial survey immediately after the pandemic, 63.7% of residents and 75% of faculty reported a reduction in technical skills. One year later, only 45.5% of residents and 21.2% of faculty members still reported skill decay, while 75% of residents and 100% of faculty were confident that residents would regain the skills necessary to eventually practice independently.

In a similar study conducted during the COVID-19 pandemic, a survey was distributed by the Royal College of Ophthalmologists to all non-retired UK members gathering information about the return to operating. Similar to what Nofi *et al* reported^[^3,4^]^, there was no objective way of quantifying the skill decay during this unexpected hiatus, so the study relies on the opinions of its subjects. It is interesting to note that the perceived anxiety, perceived increase in operating time and reduced confidence in operating ability were more prominent amongst female trainees. They were also more likely to report resource availability and to have accessed these resources including online videos and simulators, when compared with their male counterparts[15].

### Accuracy and speed

Of all the technical skills involved in surgical practice, accuracy and speed are the ones most adversely affected by time out of training. This is most clearly shown by studies on robotic VR in urology^[^6,21^]^. Participants in two studies were taught a specific simulation exercise on the robotic VR and then assessed with simulator-generated metrics. Once proficient, they stopped practicing for either 6[6] or 12[21] weeks. Upon their return, their assessment scores had deteriorated. In particular, both studies revealed quantitatively an increase in completion time, a decrease in targeting accuracy and an increase in error rate. In a real-life scenario, increases in completion times and error rates, and decreasing accuracy, could adversely affect patient safety and result in worse healthcare outcomes. These investigations reinforce the importance of minimizing skill decay prior to resuming clinical practice.

## Factors affecting the rate of skill decay

Part of the difficulty in quantifying skill decay is the fact that skills are multifaceted, each facet being affected by time out of training differently. It is therefore important to understand the factors that affect skill decay, not only to enable future research to focus on them and mitigate them individually, but also to move towards a standardized method for measuring skill decay. In 1998, Arthur *et al* compiled a comprehensive literature review, not specific to surgery, regarding factors that influence general skill decay and retention. These are also applicable to surgery and could be broadly categorized into three subheadings: (1) the complexity of the task in question, (2) the degree of overlearning or expertise of the operator prior to taking time away from the clinical environment, and (3) the retention interval itself (i.e., the length of time away and the exposure to the task, if any, during this time)[22].

### Task complexity

Intuitively, the complexity of a skill plays a role in the rate of its decay: the more complex a skill, the more liable it is to decay over a period of time. Surgical skills are no different.

Six separate studies have shown this to be true. D’Angelo *et al*[1] and Jones *et al*[14] gave pre- and post-simulation questionnaires to residents taking time out for research. The greatest reported decay was in complex procedure-specific skills that required higher levels of decision making, problem solving and technical skill^[^1,14^]^.

Bonrath *et al* found laparoscopic skill decay after 11 weeks with skills that were moderately difficult (transfer, positioning, loop tie) and difficult (intra- and extra-corporeal knot tying) but not with those that were easy (clipping, grasping, camera navigation)[23]. Maintenance of intra-corporeal knot tying after two years was also shown to degrade significantly in another study conducted by Molinas *et al*[24].

Windsor *et al* were able to quantify the different levels of skill decay for tasks of varying complexity for their novice trainees on laparoscopic simulators. The first and least complex task involved stretching a cylindrical object in one hand and then applying diathermy accurately along a predetermined line. The second task, and a more complex one, required participants to take hold of a sphere, touch it with the instrument in the opposite hand, and then apply diathermy to targets on the sphere. A score was calculated by the VR simulator which was a composite of error rate and time to complete the task. A lower score represented a better performance. Skill loss was the difference between the last score of the first training session and the first score of the second training session, divided by the last score of the first training session, and expressed as a percentage. There was a 23% loss for the standard task compared with 81% for the complex task (*P* < 0.005). Reacquisition was defined as the difference between the first and last score of the second training session, divided by the last score. Subsequently, there was a 20% reacquisition for the simple versus 54% for the complex task (*P* < 0.005)[25].

Lastly, Nofi *et al* enrolled research residents into a surgical rehabilitation program, which involved simulated laparoscopic tasks[26]. They performed similarly to level-matched clinical residents on basic tasks but required statistically significantly more time to complete suturing-based tasks (*P* < 0.001), which are often considered challenging to perform laparoscopically. Research residents gained a significant amount of confidence with cadaveric sessions (*P* < 0.05) whilst clinical residents did not. This demonstrates the need for a rehabilitation program to help surgeons maintain skills and confidence when taking time out of training.

One important caveat to these statements is that there is often no objective method of assessing task complexity. Indeed, Bonrath *et al* created a subjective scale whereby subjects themselves would rate the complexity of the task as either easy or difficult. All subjects in this task were novices so the complexity was based on their impression of a task, having never performed laparoscopic surgery. If there was a mix of answers for a task, it was classified as moderately difficult[23]. The ratings of tasks may perhaps have differed had they been more experienced in laparoscopic surgery.

### Operator-dependent factors – the art of overlearning

There are many operator-dependent factors that influence skill decay but the most important is the degree of overlearning or expertise. Overlearning refers to the deliberate overtraining of a task past a set criterion[27]. Again, this relationship appears intuitive, as the more experience surgeons have in performing a particular skill or operation, the more likely they are to retain it during a period of non-use. However, this must be caveated with the fact that more senior surgeons face higher expectations. For example, a consultant returning after time out will be expected to perform at a higher level than a registrar.

In two studies, Der *et al* and Sinha *et al*, surgical trainees were recruited to show that individuals with more experience were less affected by time out^[^6,28^]^. Perceptions closely matched objectively measured outcomes. In a third study, Schumm *et al* found that more senior trainees were more likely to report meaningful operative autonomy both at the start of the trainees’ time out for research and after a year. Though this declined for both juniors and seniors alike, seniors still perceived greater autonomy when operating[29].

### Factors associated with the retention interval

#### Length of time

Perhaps the question of most interest to management, surgeons and patients alike, is how long it takes for surgical skills to decay; with a discrete, specific answer, we could design targeted program at a certain point in time out of training to maintain surgical skills. Unfortunately, the reality is not so simple since surgical skills are multifaceted, with each facet decaying variably. Multiple studies have tried to quantify a rough estimate of the time required for skill decay to occur. Most of them have assessed laparoscopic skills, as these are easily tested and quantified using simulators. Unsurprisingly, no precise timeframe for skill decay has been found and results vary, depending on a range of factors including task complexity.

The shortest reported amount of time needed for skill decay is 11 weeks^[^23,30^]^. This was specific to difficult and moderately difficult laparoscopic skills in medical students. These tasks included extra- and intra-corporeal knot tying, transfer, positioning and loop tying. In contrast, Kahol *et al*[31] reported that, at 4 months, first year residents in laparoscopic training reported an error rate of 73%. This increased to 93% by 6 months but no *P*-value for this was provided in the research study[31]. However, Sinha *et al*[28] showed that at 6 months, only certain laparoscopic skills (presumed to be the most complex, such as clip applying and cutting) had decayed. More straightforward skills such as camera navigation, instrument navigation, camera/instrument coordination, lifting and grasping, had not decayed significantly[28]. Castellvi *et al* disagreed, stating that all laparoscopic skills required retraining at 6.5months as they all had decayed. However the degree of decay did vary between tasks[13]. Howells *et al* and Kraemer *et al* showed that at 6 months there was no significant retention of arthroscopic skills or cricothyroidotomy, respectively^[^19,32^]^. Kraemer *et al* quantified the skill retention as a percentage of subjects who achieved performance criteria: 31.8% at 6 months, 14.2% at 12 months and 60.0% at 24 months, after one refresher session[19].

The longest reported time period for skill decay included 1 year[33], 1.5 years[34] and 2 years for fine psychomotor skills[35]. At 2 years, decay was seen in all aspects of being a surgeon i.e., technical skills, clinical judgement and patient care skills[5]. On the contrary, in another investigation, a 92% retention of all tasks after 2 years was reported[36]. Despite the discrepancies in the length of time required for skill decay to occur, one conclusion is consistent: skill decay occurs to some extent for all surgical tasks regardless of the procedure.

#### Massed versus distributed practice schedule

Multiple studies have shown that distributed practice (i.e., with regular, spaced training sessions) is significantly better than massed practice (i.e., where all training sessions are completed in one sitting) in terms of retention of surgical skills^[^26,37-47^]^. However, no optimal gap between repeat sessions has been found, with studies using daily[38] or weekly[39] sessions, as well as other investigations offering training at 1 and 3 monthly interval[40] or at 2 weeks, 4 weeks and 6 months[41]. Interestingly, Mitchell *et al* found no difference between spaced repetitions in which participants practiced one session per week for 4 consecutive weeks, as opposed to one session per month for 4 consecutive months[42]. This may be because neither of these strategies is high-frequency, low-intensity and thus, depending on the type of skills being taught, both may be suboptimal.

There is also one significant limitation to all of these studies, and indeed the majority of those included in this review, which is the sample size. Of the investigations that evaluate massed versus distributed practice, the largest sample size is 145 medical students[38]. However, when considering that this sample is then subdivided into six different groups which undergo different training, each group only contains 24 participants. Indeed, the individual subgroups in all of these papers vary from 9 participants[40] to at most 24 participants[38]. With such small sample sizes, it is difficult to draw reliable, statistically significant conclusions.

## Strategies to mitigate technical skill decay

Much of the research performed in the area of skill decay involves the use of simulation in robotic and laparoscopic surgery. Simulators allow for close monitoring of factors that may influence the measurement of skill decay and also allow for consistent measurements of skills.

Simulation is an all-encompassing term – from box simulators to VR as well as cadaveric dissections^[^1,2,10,13,15,20,23,26,40,41^]^. All have been shown to play a role in reducing the rate of skill decay and allow for maintenance of skills when a surgeon is experiencing a period of minimal case-loads^[^10-12^]^. In one study, 15 novices were randomized to train on a laparoscopic simulator or to a control group that had no training. Both groups were then assessed on the simulator and on a live porcine laparoscopic Nissen fundoplication model at training completion and 5 months later. Performance of the trained individuals unsurprisingly outperformed controls. Whilst this performance deteriorated slightly at retention at 5 months, this was not the case with the live porcine model showing durable improvement in operative skill of trainees[48]. Also, when skill decay has already occurred, simulation allows for a safe model for reacquisition of skills and confidence prior to interaction with patients^[^13,26^]^. Some simulation-based educational strategies may be more effective than others in reducing skill decay – in one such study, video-trainers resulted in improved retention of skills than VR simulators[20]. However, this has not been extensively researched in other investigations.

Alongside the use of simulation, a plausible additional method to attenuate skill decay is to provide research residents with the opportunity to “moonlight” in order to remain clinically active^[^1,2^]^. According to a survey conducted by Jamshidi and colleagues[49], beside the financial incentive, research residents stated that their second motivation for undertaking moonlighting was to maintain their clinical acumen. In D’Angelo’s *et al* study[1], research residents who performed surgical operations during on-call responsibilities reported a higher pre-procedure and post-procedure confidence for the bowel anastomosis and LVH repair tasks; however, no such correlation was demonstrated with regards to urinary catheterization and subclavian line insertion[1]. It is also worth noting that not all moonlighting experiences include operating and some principal investigators may be reluctant in allowing research students to be distracted by this added work[2].

When specifically researching methods to mitigate technical skill decay, one theoretical paper on cognitive training for the prevention of skill decay was found[50]. It describes methods including ideomotoric training, whereby movement patterns are visualized and verbalized or subvocal imagery, in which internal speech conjures visual imagery^[^50,51^]^. Though practical training was still found to show the greatest improvement in a task-specific checklist, mental training did show an additional benefit. The extent to which mental training improves performance remains unclear[51]. Another useful training adjunct is a video with a voiceover recorded for each phase of the procedure. One such training tool was developed as the Imperial Knee Arthroscopy Cognitive Task Analysis tool and implemented in a randomized controlled trial. Participants found this to be a useful adjunct to learning in the operating room[52].

Multiple studies have called for the requirement of a surgical “bootcamp” prior to return to work after a period out of training^[^2,26^]^. Further research is needed to elucidate the precise curriculum for this program.

## Discussion

This review assesses the existing evidence on the impact of time away from surgical practice on surgical skills. It reveals nuanced insights into the facets most susceptible to deterioration, the factors contributing to skill decay, and the potential role of simulation in both maintaining and reacquiring surgical proficiency.

The first striking aspect is the paucity of research articles directly related to this topic and the small sample sizes. The majority of studies included (83%, 34/41) had fewer than 50 participants, rendering the statistical significance of some results questionable. This is likely due to the limited understanding of surgical skill decay, and the difficulty in its quantification. Currently, there is no globally accepted way to quantify performance. Therefore, no methods are available to quantitatively assess the degree of training a surgeon has received or their baseline performance, prior to time out. Similarly, there are no tools to quantitatively assess the post-time out of training performance to identify surgeons who require retraining as they are no longer able to perform to an adequate level. These key pillars must first be determined in order to answer our question reliably.

Despite these limitations, the research demonstrates that the skills that are most adversely affected by time out of training are technical operative skills and, within those, speed and accuracy in the operations. These skills are vital to maintaining a high level of patient safety. Accordingly, the findings demonstrate the potential for a structured return-to-work program that focusses on the reacquisition of operating skills.

Importantly, it seems that female trainees are more likely to be affected by time out of training in terms of their perception of their operative ability and their confidence regarding returning to the operating theatre. Surgery continues to be a male-dominated specialty so one way of rectifying this imbalance is to address female trainees concerns and provide them with the necessary support to succeed. This is especially important as women who want a family will need to take time away from their clinical practice for maternity leave.

The next important question is the factors that affect skill decay. These can be broadly categorized into three classes, namely those associated (1) with the task itself, (2) with the operator and (3) with the retention interval. In sum, complex tasks performed by novices after a long retention interval with only massed practice at the start will show the largest degree of skill decay. The extent to which these factors individually contribute to skill decay is unclear. However, in the design of a structured return-to-work program, each should be addressed and optimized to increase the chances of success in minimizing skill decay.

Lastly, simulation-based training emerges as a cornerstone in combating skill decay and facilitating the maintenance and reacquisition of surgical proficiency. High-fidelity simulations offer a controlled environment for surgeons to practice and refine their skills without compromising patient safety. From novice to experienced surgeons, simulation-based interventions bridge the gap between theory and practical application, fostering continuous learning and providing opportunities to adapt to novel procedures and technologies. Moreover, simulators tailored to mimic real surgical scenarios aid in restoring degraded skills after periods of inactivity, facilitating a swift return to optimal performance.

In the ever-evolving landscape of surgical practice, acknowledging the vulnerable aspects of skill decay and its influential factors is imperative for sustaining high-quality patient care. The evidence underscores the critical need for interventions aimed at mitigating skill decay. Simulation-based training emerges not only as a preventative measure but also as a restorative tool to counteract the effects of prolonged absence from surgical practice. Embracing simulation-based interventions within continuous professional development frameworks can be pivotal in the preservation, enhancement and swift reacquisition of surgical skills.

## Conclusion

Skill decay is encountered by the majority of surgical trainees taking time out of training to pursue academic research, higher degrees, parental leave, or other interests. There is no doubt that it occurs and has an impact on their ability to return to work safely, confidently and at the same operative level. Nevertheless, the degree of skill decay and the most effective methods of mitigation are questions that remain largely unanswered.

There is room for considerable research in this area including:

- An international definition of skill decay as applied to surgery.
- Evidence-based quantification of performance – to reliably assess baseline performance and post-time out of training performance, including the exploration of the role that Artificial Intelligence may play in this field and its potential future application.
- Quantification of skill decay mitigation strategies including different forms of simulation.
- Development of a structured program specifically targeting operative skills and the impact of this on confidence and operative ability to support trainees back into program after time out.

Despite these challenges, it is likely that the importance of skill decay will become increasingly prominent in surgical training and that simulation will be used as a method of mitigation. As such, further research into this area should be encouraged as it will be of the utmost importance in ensuring a continued high standard of surgical care.

## Data Availability

Not applicable.

## Research registration unique identifying number (UIN)

Research registry UIN 11031 from https://www.researchregistry.com/browse-theregistry#home/registrationdetails/67ad9922054efd0306f78c7b/).
